# Appointment of Dr. Hunt as Surgeon to the U. S. N.

**Published:** 1863-01

**Authors:** 


Appointment of Dr. Hunt as Surgeon to the U. S. N.—Dr. S. B. Hunt of this City, has recently received appointment as Surgeon to the U. S. N. He has been a resident of Buffalo for the last ten years, and is distinguished for his literary and scientific attainments. He is widely known as a medical scholar and writer; and for several years was editor of the *Buffalo Medical Journal*, and by the able and impartial manner in which he conducted it, as well as by the valuable articles he contributed to its pages, earned imperishable honors, and at the same time made his Journal one of the best and most instructive in this country. He was also Professor of Anatomy in the University of Buffalo, and as a student and teacher in this branch of medical knowledge is unsurpassed.

While we regret that so distinguished a medical and popular author should remove from the City, we yet most heartily congratulate the Doctor, and especially the Naval service upon his appointment.

